# Effect of high-frequency oscillatory ventilation on esophageal and transpulmonary pressures in moderate-to-severe acute respiratory distress syndrome

**DOI:** 10.1186/s13613-016-0181-1

**Published:** 2016-08-30

**Authors:** Christophe Guervilly, Jean-Marie Forel, Sami Hraiech, Antoine Roch, Daniel Talmor, Laurent Papazian

**Affiliations:** 1 Aix-Marseille Univ, APHM, URMITE UMR CNRS 7278, Hôpital Nord, Réanimation des Détresses Respiratoires et Infections Sévères, Marseille, France; 2 Service d’Accueil des Urgences, APHM, Hôpital Nord, Marseille, France; 3 Department of Anesthesia, Critical Care, and Pain Medicine, Beth Israel Deaconess Medical Center, 330 Brookline Ave, Boston, MA USA

## Abstract

**Background:**

High-frequency oscillatory ventilation (HFOV) has not been shown to be beneficial in the management of moderate-to-severe acute respiratory distress syndrome (ARDS). There is uncertainty about the actual pressure applied into the lung during HFOV. We therefore performed a study to compare the transpulmonary pressure (*P*_L_) during conventional mechanical ventilation (CMV) and different levels of mean airway pressure (mPaw) during HFOV.

**Methods:**

This is a prospective randomized crossover study in a university teaching hospital. An esophageal balloon catheter was used to measure esophageal pressures (Pes) at end inspiration and end expiration and to calculate *P*_L_. Measurements were taken during ventilation with CMV (CMVpre) after which patients were switched to HFOV with three 1-h different levels of mPaw set at +5, +10 and +15 cm H_2_O above the mean airway pressure measured during CMV. Patients were thereafter switched back to CMV (CMVpost).

**Results:**

Ten patients with moderate-to-severe ARDS were included. We demonstrated a linear increase in Pes and *P*_L_ with the increase in mPaw during HFOV. Contrary to CMV, *P*_L_ was always positive during HFOV whatever the level of mPaw applied but not associated with improvement in oxygenation. We found significant correlations between mPaw and Pes.

**Conclusion:**

HFOV with high level of mPaw increases transpulmonary pressures without improvement in oxygenation.

## Background

Moderate or severe acute respiratory distress syndrome (ARDS) [1] is associated with substantial mortality. Use of a lung-protective strategy with low tidal volume (*V*_t_) of 6 ml/kg of predicted body weight has been associated with improved outcomes [2]. High-frequency oscillatory ventilation (HFOV) is a non-conventional mode which has been proposed to achieve the targets of protective ventilation with very low *V*_t_ [3] and a greater alveolar stability due to relatively constant mean airway pressure (mPaw) [4]. However, two large recently published randomized clinical trials, OSCAR [5] and OSCILLATE [6], failed to prove any clinical benefit when HFOV was applied in adults with moderate-to-severe ARDS as compared with a strategy with low tidal volume, high positive expiratory pressure (PEEP) and limited plateau pressure (Pplat). In the latter study, side effects of HFOV were observed with more requirements for vasopressors, likely due to right ventricular failure secondary to high mPaw used [7, 8].

Another possible explanation of the lack of clinical benefit with HFOV in adults with ARDS may be due to the occurrence of pulmonary overdistension in non-dependant areas of the lung [9]. Because mPaw during HFOV does not reflect of the real pressure applied to the alveoli [10], with non-predictable attenuation all along the trachea–bronchial tree, it is not possible to know the true pulmonary distending pressure. Esophageal pressure (Pes) is an approximation of the pleural pressure, and its use has shown a possible clinical benefit when PEEP was set according to the value of Pes in moderate-to-severe ARDS [11]. Esophageal pressure measurement allows the calculation of the maximal and minimal transpulmonary pressures (*P*_L_) applied during mechanical ventilation. Data reporting *P*_L_ during HFOV are scarce [12] and only describe the feasibility of the technique but not the comparison of range of *P*_L_ occurring during the switch from CMV to an HFOV trial. Therefore, we performed a prospective study of *P*_L_ determination in moderate-to-severe ARDS during and after an HFOV trial.

## Methods

This is an ancillary study of a previously published study [7].

### Patients

The study was approved by the ethics committee of the Marseille University Hospital (Comité de Protection des Personnes Sud Méditerranée, ID RCB:2008-A00077-48). Written informed consent was obtained from each patient’s next of kin. Patients admitted in the intensive care unit of a university teaching hospital during a 10-month period were screened if they met inclusion criteria: moderate-to-severe ARDS with a PaO_2_/FiO_2_ ratio ≤150 mmHg at a PEEP ≥8 cm H_2_O. Exclusion criteria were age <18 years, moribund status, risks associated with HFOV (head injury, pneumothorax or a chest tube in place with persistent air leak) and contraindications to the placement of a nasogastric probe. All patients were sedated and continuously paralyzed [13]. The severity of illness was determined according to the Simplified Acute Physiologic II Score, the Sepsis-related Organ Failure Assessment Score and the Lung Injury Score [14, 15].

### Tested ventilatory strategies

**Fig. 1:**
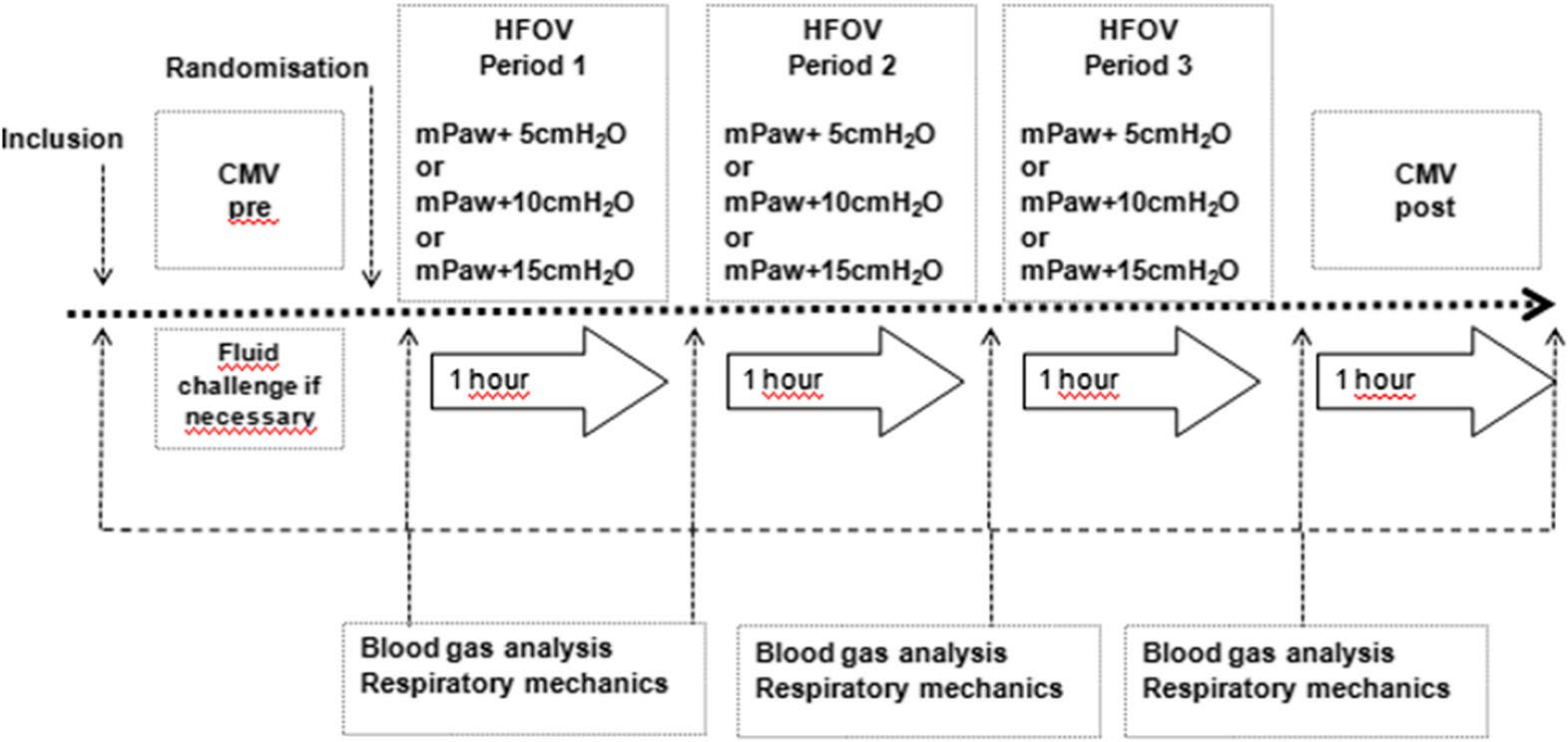
Study design

Patients were submitted to a 6-h period of CMV (CMVpre) in volume-controlled, constant square flow, mode using the AVEA ventilator (VIASYS Healthcare, Palm Springs, CA, USA) with a tidal volume of 6 mL/kg of predicted body weight adjusted to obtain a plateau pressure <30 cm H_2_O. PEEP and FiO_2_ were adjusted according to the ARMA protocol [2]. Patients were then switched to HFOV using a 3100B ventilator (SensorMedics, Yorba Linda, CA, USA). After a recruitment maneuver was performed with a mPaw of 40 cm H_2_O during 40 s with a pressure amplitude of oscillation of 0 cm H_2_O [16], HFOV was set as follows: FiO_2_ as during the CMVpre period; frequency of 5 Hz; inspiratory time of 33 %; and bias flow of 40 L/min. The pressure amplitude of oscillation and frequency were then adjusted to achieve a PaCO_2_ close to the PaCO_2_ measured during the CMVpre period. If pressure amplitude of oscillation of 110 cm H_2_O was insufficient to achieve a pH ≥ 7.25, frequency was decreased at 4 Hz. The protocol consisted of three 1-h periods of HFOV (HFO + 5, HFO + 10, HFO + 15) in a randomized order, with a mPaw level calculated by adding 5, 10 or 15 cm H_2_O to the mPaw measured during the CMVpre period (Fig. 1). A recruitment maneuver was performed at the beginning of each HFOV period and before switch back to CMV. Respiratory frequency and pressure amplitude of oscillation were adjusted to maintain PaCO_2_ constant during the protocol. Measurements were taken at the end of each period of the protocol and 1 h after switch back to CMV. During the protocol, norepinephrine infusion was adjusted to maintain a mean arterial pressure above 65 mmHg.

### Esophageal and transpulmonary pressure measurements

A specific nasogastric feeding probe (SmartCath^®^, VIASYS Healthcare, Palm Springs, CA, USA) equipped with an esophageal balloon was inserted after in vitro automatized test for leak search and compliance measurement, and then the balloon was filled with a volume of air between 0.5 and 2 mL as recommended by the manufacturer. Every 30 min, the ventilator evacuates and refills the balloon to maintain measurement accuracy. The correct positioning in the lower third of the esophagus was confirmed by the presence of cardiac artifacts, the changes in transpulmonary pressure during tidal ventilation and the parallelism of airway and esophageal curves after the interruption of a brief chest compression maneuver [17]. Finally, a chest X-ray excluded the misplacement of the probe into the airway. Esophageal pressures were recorded and monitored by the integrated system, CP-100 pulmonary monitor (Bicore Monitoring System Inc^®^, Irvine, CA, USA) present in the AVEA ventilator. An end-inspiratory occlusion of 2 s allowed the measurement of, respectively, Pplat and inspiratory Pes (Pes_insp_), whereas an end-expiratory occlusion of 5 s allowed the measurement of, respectively, total PEEP (PEEPtot) and expiratory Pes (Pes_exp_) during CMV. During HFOV periods, because interruption of ventilation is not possible, screen of the AVEA ventilator was frozen for measuring the peak and trough amplitude of oscillations for measurements of, respectively, the maximum and minimum Pes. The following formulas were computed as follows:

$$\begin{aligned} & {\text{PEEP}}_{\text{tot}} {\text{ = external}}\,{\text{PEEP}} + {\text{intrinsic}}\,{\text{PEEP}} \\ & {\text{Driving}}\,{\text{pressure}} = {\text{Pplat}} - {\text{PEEP}}_{\text{tot}} \\ \end{aligned}$$

During CMV,

$$\begin{aligned} & P_{\text{Linsp}} = {\text{Pplat}} - {\text{Pes}}_{\text{insp}} \\ & P_{\text{Lexp}} = {\text{PEEP}}_{\text{tot}} - {\text{Pes}}_{ \exp } \\ & {\text{Respiratory}}\,{\text{system}}\,{\text{elastance }}\left( {{\text{EL}}_{\text{RS}} } \right) \, = \, \left( {{\text{Pplat}}{-}{\text{PEEP}}_{\text{tot}} } \right)/V_{\text{t}} \\ & {\text{Chest}}\,{\text{wall}}\,{\text{elastance}}\,\left( {{\text{EL}}_{\text{CW}} } \right) = \, ({\text{Pes}}_{ \rm{insp} } - {\text{Pes}}_{ \rm{exp} } )/V_{\text{t}} \\ & {\text{Pulmonary}}\,{\text{elastance}}\,\left( {{\text{EL}}_{\text{L}} } \right) = {\text{EL}}_{\text{RS}} - {\text{EL}}_{\text{CW}} = \, (P_{\text{Lmax}} - \, P_{\text{Lmin}} )/V_{\text{t}} \\\end{aligned}$$

During HFOV,

$$\begin{aligned} & {\text{Pes}}_{\rm{mean}} = \left( {{\text{Pes}}_{ \rm{max} } + {\text{Pes}}_{\rm{min} } } \right)/2 \\ & P_{{L{\text{mean}}}} {\text{ = mPaw}} - {\text{Pes}}_{\text{mean}} \\ \end{aligned}$$

**Fig. 2:**
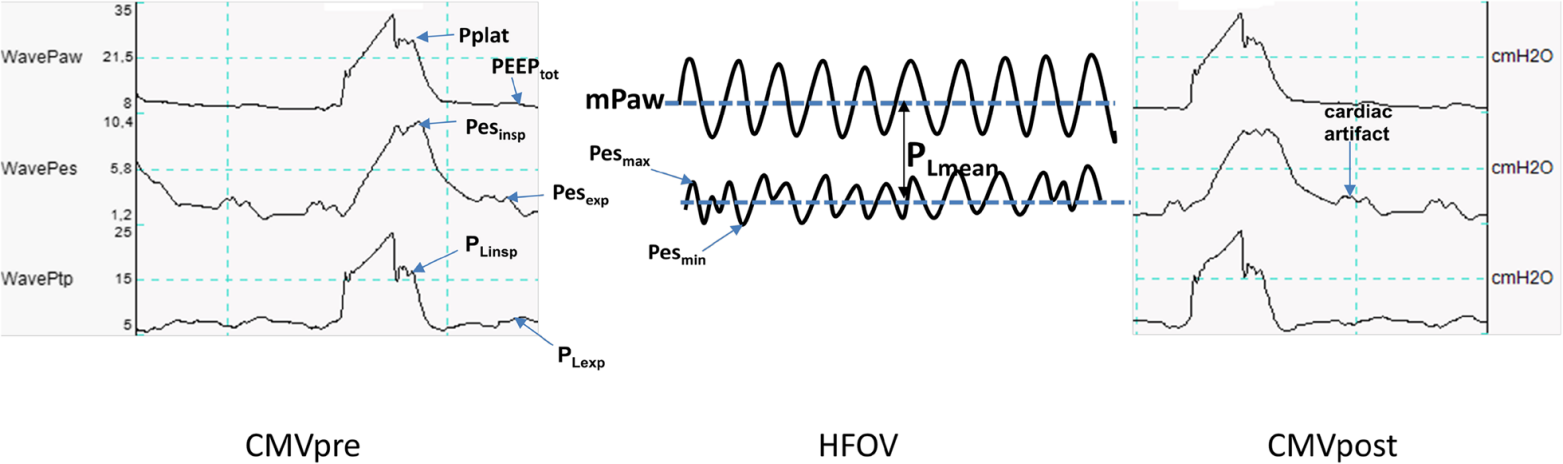
Representative tracings of mean airway pressure (mPaw), plateau pressure (Pplat), total positive expiratory pressure (PEEP_tot_), esophageal pressure (Pes) and transpulmonary pressure (*P*_L_) during conventional mechanical ventilation (CMV) and high-frequency oscillatory ventilation

An example of tracings in the two ventilatory modes with the airway, esophageal and transpulmonary pressures determinations and calculations is provided in Fig. 2.

### Statistical analysis

Data are presented as mean ± SD or median (interquartile range) as required. Normality of variables was tested according the Kolmogorov–Smirnov test. Repeated-measures analysis of variance or Friedman’s test was used to evaluate the effect of time and mPaw level. The Tukey test or the Wilcoxon test was used for intergroup comparisons. Bivariate correlations with Spearman’s test for each period of ventilation were performed. All statistics and figures were performed with the SPSS 20.0 package (SPSS, Chicago, IL, USA).

## Results

Among the 16 patients included in the princeps study [7], ten patients were monitored by the esophageal catheter and were used in this study.

Table 1 reports patient’s characteristics at inclusion. Initial computed tomography scan or thoracic radiograph showed five lobar and five diffuse presentations. Causes of ARDS were bacterial pneumonia (*n* = 4), influenza A (H1N1), (*n* = 2), aspiration (*n* = 2), post-cardiopulmonary resuscitation (CPR) (*n* = 1) and acute pancreatitis (*n* = 1). Fluid loading was performed in three patients during the CMVpre period. At baseline, all except one received norepinephrine infusion to maintain mean arterial pressure (MAP) above 65 mmHg.

**Table 1:** Patient characteristics and respiratory data at inclusion

Age (years)	63 ± 15
Gender (male), n (%)	4 (40)
Body mass index (kg/m^2^)	29 ± 8
SAPS II at the admission	49 ± 23
SOFA at the admission	11 ± 3
ICU mortality, *n* (%)	4(40)
Direct lung injury, *n* (%)	9 (90)
CT scan or X-ray presentation (lobar/diffuse), *n*	5/5
PaO_2_/FiO_2_ ratio (mmHg)	131 ± 51
FiO_2_	0.74 ± 0.17
PaCO_2_ (mmHg)	46 ± 7
PEEP (cm H_2_O)	13 ± 3
*V*_t_ (mL)/(mL/kg/IPBW)	382 ± 41/6.6 ± 0.7
Respiratory rate (cycle/min)	26 ± 4
Plateau airway pressure (cm H_2_O)	24 ± 4
Driving pressure (cm H_2_O)	12 ± 3
mPaw (cm H_2_O)	18 ± 3
Oxygenation index	17 ± 9
Lung Injury Score at the inclusion	3.0 ± 0.5
Time from ARDS to inclusion (*d*)	0 ± 0.5

Oxygenation index was calculated as mean airway pressure × FiO_2_ × 100)/PaO_2_. Results are provided as mean ± SD

FiO_2_, inspired oxygen fraction; PEEP, positive end-expiratory pressure; mPaw, mean airway pressure; IPBW, ideal predicted body weight; SAPS II, Simplified Acute Physiology Score II; SOFA, Sepsis Organ Failure Assessment Score; *V*_t_, tidal volume

### Respiratory parameters

During HFOV, mPaw was progressively increased from 18 ± 4 cm H_2_O in CMVpre period to 33 ± 4 cm H_2_O at HFO + 15 (Table 2). PaO_2_/FiO_2_ ratio did not significantly change under HFOV when compared with the CMVpre period. However, it increased by more than 20 % in three patients at HFO + 5, in four patients at HFO + 10 and in two patients at HFO + 15. FiO_2_ was slightly lower at the end of the study. Worsening of oxygenation occurred in two patients at HFO + 5, in three patients at HFO + 10 and in four patients at HFO + 15. As required by the protocol, PaCO_2_ and pH were kept constant throughout the study. Concerning respiratory mechanics, Pplat, driving pressure, respiratory system elastance, chest wall elastance and pulmonary elastance were similar during the CMVpre and CMVpost periods. These last parameters could not be calculated during the HFOV periods because of the lack of tidal volume monitoring.

**Table 2:** Gas exchanges and respiratory mechanics

	CMVpre	HFO + 5	HFO + 10	HFO + 15	CMVpost	*p* value time
mPaw (cm of H_2_O)	18 ± 4^a,b,c^	23 ± 4^b,c,d,e^	28 ± 4^a,c,d,e^	33 ± 4^a,b,d,e^	17 ± 4^a,b,c^	*<0.001*
PaO_2_/inspired O_2_ fraction (mmHg)	131 ± 51	132 ± 56	125 ± 23	138 ± 49	139 ± 34	0.9
Inspired O_2_ fraction	74 ± 17	71 ± 16	72 ± 16	77 ± 18	67 ± 10^d^	*0.03*
Arterial pH	7.29 ± 0.04	7.31 ± 0.09	7.31 ± 0.01	7.29 ± 0.1	7.32 ± 0.06	0.3
PaCO_2_ (mmHg)	46 ± 7	47 ± 12	46 ± 14	46 ± 9	42 ± 7	0.5
PEEP (cm of H_2_O)	13 ± 3	NA	NA	NA	12 ± 3	0.4
*V*_t_ (ml/kg)	6.6 ± 0.7	NA	NA	NA	6.7 ± 0.8	0.2
Plateau airway pressure (cm of H_2_O)	24.5 ± 4	NA	NA	NA	23.5 ± 4	0.06
Driving pressure (cm of H_2_O)	11.8 ± 3.4	NA	NA	NA	11.5 ± 3.3	0.4
Power of oscillations, %	NA	73 ± 23	79 ± 29	81 ± 24	NA	0.1
Respiratory rate (cycle/min)	26 ± 4	NA	NA	NA	25 ± 6	1
Oscillatory frequency (Hz)	NA	4.8 ± 1	4.7 ± 0.7	4.6 ± 1	NA	0.2
Inspiratory esophageal pressure	15 [11.5; 21.2]	NA	NA	NA	14 [10.2; 17.2]	0.1
Expiratory esophageal pressure	12.5 [5.1; 13.5]	NA	NA	NA	9.1 [5.4; 13.5]	0.1
Mean esophageal pressure (cm of H_2_O)	NA	12.4 [10.6; 16.7]^b,c^	16.7 [12.5; 18.7]^a,c^	19.1 [16.7; 23.3]^a,b^	NA	*0.001*
Inspiratory *P*_L_ (cm of H_2_O)	8.1 [5.7; 12.8]	NA	NA	NA	11.8 [5; 12.1]	1
Expiratory *P*_L_ (cm of H_2_O)	−1 [−3; + 0.7)	NA	NA	NA	+3.5 [−3; + 6]	0.2
Mean *P*_L_ (cm of H_2_O)	NA	10.5 [7.3; 13.8]^c^	13.1 [9.2; 14.7]	14 [11.5; 16.3]^a^	NA	*0.001*
Respiratory system elastance (cm of H_2_O/L)	31.2 ± 9.7	NA	NA	NA	29.9 ± 8	0.2
Chest wall elastance (cm of H_2_O/L)	15.7 ± 6	NA	NA	NA	11.1 ± 4.5	0.3
Pulmonary elastance (cm of H_2_O/L)	15.9. ± 11	NA	NA	NA	18.9 ± 6	0.3

*P*_L_ transpulmonary pressure, *NA* not applicable

*p* values in italic are provided for < 0.05

^a^p < .05 vs. HFO + 5, ^b^ *p* < .05 vs. HFO + 10, ^c^ *p* < .05 vs. HFO + 15, ^d^ *p* < .05 vs. CMVpre, ^e^ *p* < .05 vs. CMVpost

**Fig. 3:**
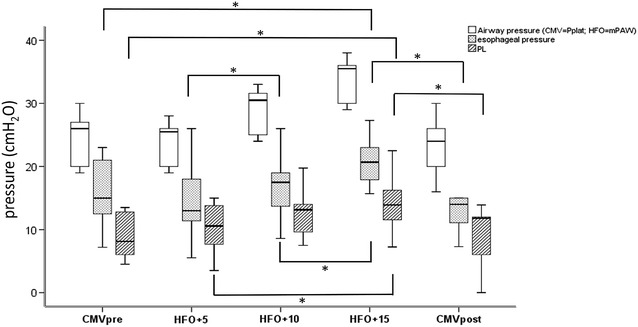
Airway pressures, esophageal pressures and transpulmonary pressures (*P*_L_) during conventional mechanical ventilation (CMV) before and after three levels of mean airway pressure (mPaw) during high-frequency oscillatory ventilation (HFO). Airway pressure is plateau pressure (Pplat) during CMV and mPaw during HFO. Esophageal pressure is the inspiratory pressure during CMV and the mean esophageal pressure during HFO. *P*_L_ is computed by Pplat minus inspiratory esophageal pressure ding CMV and mPaw minus mean esophageal pressure during HFO. *Means < 0.05

At similar level of mPaw, during the volumetric periods of ventilation (CMVpre and CMV post), we did not find differences concerning esophageal and transpulmonary pressures. During HFOV periods, we observed a linear increase in esophageal pressure from 12.4 [10.6; 16.7] to 19.1 [16.7; 23.3] cm of H_2_O (*p* = 0.001) as mPaw increases from 23 ± 4 to 33 ± 4 cm of H_2_O. As a consequence, mean *P*_L_ increased during HFOV periods from 10.5 [7.3; 13.8] to 14 [11.5; 16.3] cm of H_2_O (Table 2; Fig. 3). Interestingly, there was no negative transpulmonary pressure whatever the period of HFOV ventilation. There were, however, seven (out of ten) patients with negative minimal *P*_L_ during the CMVpre period and only three (out of ten) patients during the CMVpost period (*p* = 0.07, *χ*^2^ test).

During HFO, mPaw was correlated with Pes_mean_ at HFO + 5 and HFO +15 periods (respectively, *ρ* = 0.71, *p* = 0.02 and *ρ* = 0.84, *p* = 0.02) but at no time with *P*_*L*mean_.

## Discussion

The present study assessing the use of esophageal pressure measurements in patient with moderate-to-severe ARDS on whom a trial of HFOV is performed demonstrates (1) a linear increase in transpulmonary pressures with the increase in mPaw during HFOV, (2) a minimal transpulmonary pressure which was always >0 during HFOV and (3) a correlation between mean esophageal pressure and mPaw.

For decades, HFOV has been used for respiratory failure in both adults and children who were inadequately responsive to conventional mechanical ventilation. However, recently the results of the OSCAR [5] and OSCILLATE [6] studies performed on adults have not shown benefit to HFOV over conventional ventilation. A recent study in the pediatric population has also shown equivocal results with HFOV [18]. Indeed, positive studies on HFOV are limited [16, 19] and predate the era of low tidal volume conventional mechanical ventilation. During HFOV, there is uncertainty about the real pressure that is applied to the alveoli and therefore the distending pressure applied into the lung. Henderson et al. [12] have previously described the use of esophageal manometry to measure *P*_L_ during HFOV. With a mean airway pressure of 27 ± 5 cm H_2_O during HFOV, they measured a mean esophageal pressure of 14 ± 4 cm H_2_O and computed a mean *P*_L_ of 18 ± 4 cm H_2_O. These data are consistent with the present results, namely a mPaw of 28 ± 4 cm H_2_O, results in a median of 16.7 IQR [12.5; 18.7] cm H_2_O range of Pes and a median of 13.1 IQR [9.2; 14.7] cm H_2_O range of *P*_L_. The safe range of *P*_L_ during HFO is not known. However, during conventional mechanical ventilation for ARDS, a *P*_L_ > 27 cm H_2_O is associated with an unacceptably high level of strain [20]. The *P*_L_ value recorded during HFO remains below this threshold whatever the level of mPaw.

One interesting result is the correlation between mPaw and esophageal pressure that we obtained; the more mPaw is set, the more Pes is measured. In clinical practice, levels of mPaw in the OSCAR and OSCILLATE trials [5, 6] were not exactly the same. During the first 2 days of the studies, mPaw was set at 5 cm H_2_O higher in the Canadian trial than in the UK trial. These differences could have led to more pulmonary overdistension and side effects that could explain the deleterious outcomes observed with HFOV in the OSCILLATE trial.

An ongoing study, the EPOCH study [21], which aim is to compare a strategy of preventing atelectrauma with a *P*_L_ of 0 cm H_2_O at end expiration to a strategy of lung recruitment to target *P*_L_ of 15 cm H_2_O at end-inspiratory volume in a crossover design either with CMV and either with HFOV will clarify the protective or deleterious roles of HFOV as compared to CMV.

### Limitations

First, as measurements of esophageal pressure could not be taken in static conditions during HFO periods, we cannot rule out a possible bias of measurements due to cardiac artifacts. However, the use of mean esophageal pressure reduces this bias. Second, during HFOV, due to the lack of *V*_t_ monitoring, we use the calculation of *P*_L_ derived from Pes measurements [22] and not the elastance-derived measurements of *P*_L_ [23] which could lead to different results [24]. Indeed, experimental data have shown that although recorded value of Pes is a quite accurate approximation of measured pleural pressure in the middle part of the lungs, Pes can overestimate or underestimate the value of pleural pressure whether in the non-dependant part and whether in the dependant part of the lungs [25]. The more convincing results are that the variations of Peso reflect those in pleural pressure whatever the parts of the lung [26]. There is still a matter of controversy on the use of the former or the latter method. A prospective ongoing study could bring a response to the clinical utility of the method used [27]. Third, because we have not performed the registration of airway pressure during HFO, we cannot exclude negative *P*_L_ during the active expiratory phase, and further studies are needed to conclude. And fourth, from a technical point of view, we also cannot exclude that larger inflation volume as demonstrated by Mojoli et al. [28] could have led to different results. However, our study precedes the one from Mojoli, and we used the volume and the proceeding recommended by the manufacturer.

We cannot speculate whether lower mPaw during HFOV, the same range as recorded in CMV, could lead to lower esophageal and transpulmonary pressures recorded. A level of <+10 cm H_2_O of mPaw during HFO does not increase significantly Peso and *P*_L_. Only a level of ≥+15 cm H_2_O of mPaw increases significantly both Peso and *P*_L_.

## Conclusion

The use of high mean airway pressures during HFOV leads to increase in transpulmonary pressures. Contrary to CMV, during HFOV, transpulmonary pressure remains always positive.
